# Aluminum Exposure in Neonatal Patients Using the Least Contaminated Parenteral Nutrition Solution Products

**DOI:** 10.3390/nu4111566

**Published:** 2012-11-02

**Authors:** Robert L. Poole, Kevin P. Pieroni, Shabnam Gaskari, Tessa Dixon, John A. Kerner

**Affiliations:** 1 Department of Pharmacy, Lucile Packard Children’s Hospital at Stanford, 725 Welch Road, Palo Alto, CA 94304, USA; Email: SGaskari@lpch.org (S.G.); Tessarx@gmail.com (T.D.); 2 Division of Gastroenterology, Department of Pediatrics, Hepatology, and Nutrition, Stanford University School of Medicine, 750 Welch Road, Suite 116, Palo Alto, CA 94304, USA; Email: Pieronifive@yahoo.com (K.P.P.); JKerner@stanfordmed.org (J.A.K.)

**Keywords:** aluminum, parenteral nutrition, contamination, neonates, toxicity

## Abstract

Aluminum (Al) is a contaminant in all parenteral nutrition (PN) solution component products. Manufacturers currently label these products with the maximum Al content at the time of expiry. We recently published data to establish the actual measured concentration of Al in PN solution products prior to being compounded in the clinical setting [1]. The investigation assessed quantitative Al content of all available products used in the formulation of PN solutions. The objective of this study was to assess the Al exposure in neonatal patients using the least contaminated PN solutions and determine if it is possible to meet the FDA “safe limit” of less than 5 μg/kg/day of Al. The measured concentrations from our previous study were analyzed and the least contaminated products were identified. These concentrations were entered into our PN software and the least possible Al exposure was determined. A significant decrease (41%–44%) in the Al exposure in neonatal patients can be achieved using the least contaminated products, but the FDA “safe limit” of less than 5 μg/kg/day of Al was not met. However, minimizing the Al exposure may decrease the likelihood of developing Al toxicity from PN.

## 1. Introduction

Aluminum is the most abundant metallic element on Earth and is naturally occurring in certain minerals, ores, oxides, and silicates. Humans are exposed to aluminum on a daily basis through drinking water, food, medications, dust, and deodorants. Aluminum serves no biologic function in humans and in a healthy individual, aluminum exposure causes little harm due to poor oral bioavailability. The gastrointestinal tract allows less than 1% of ingested aluminum into the blood stream. Renal excretion then removes 99% of the aluminum that enters the blood stream [2]. Despite these protective mechanisms, aluminum toxicity has been documented in the medical literature for over 30 years [2,3,4,5,6,7,8,9]. Recorded manifestations of aluminum toxicity include fracturing osteomalacia and reduced bone mineralization, neurological dysfunction and dialysis encephalopathy, microcytic hypochromic anemia, and cholestasis. Parenteral nutrition (PN) has long been implicated as a major source of aluminum exposure due to contamination of the component ingredients. PN component products are contaminated with aluminum in raw materials as well as byproducts from the manufacturing process, where aluminum leaches from glass vials during autoclaving [10,11,12]. Patients at greatest risk for aluminum toxicity from PN include those with underlying renal dysfunction and prolonged courses of PN therapy. Premature infants are particularly at high risk of aluminum accumulation and toxicity as they often require PN for many days and have immature kidneys incapable of excreting aluminum efficiently. Calcium gluconate and phosphate salts are known to be especially high in aluminum content and are often administered to premature infants in substantial amounts to promote bone mineralization [10,13,14]. 

In an attempt to limit the risk of aluminum toxicity, the U.S. Food and Drug Administration (FDA) modified its “Regulations on Aluminum in Large and Small Volume Parenterals Used in Total Parenteral Nutrition” when the January 2000 Final Rule was enacted in July 2004 [15,16]. The Final Rule limits the aluminum concentration of large volume parenteral products to 25 μg/L. Small volume parenteral products must state the maximum aluminum concentration at the time of product expiry on the manufacturer’s product label. However, there is no maximum aluminum concentration label requirement for small volume parenterals. Manufacturers of all PN products must also include a package insert with a standard warning describing the presence of aluminum in the product; the risk of using the products in infants and patients with impaired kidney function; and a recommended maximum daily aluminum dose of 4 to 5 μg/kg/day to prevent accumulation and toxicity.

Our group recently published data quantifying the actual aluminum concentration of all available PN solution products in the United States [1]. Our data documented that the actual aluminum concentration in PN solution products is significantly less than labeled. The objective of this study is to assess the aluminum exposure in neonatal patients using the least contaminated PN solutions and determine if it is possible to meet the FDA recommended maximum daily aluminum exposure of 4 to 5 μg/kg/day. 

## 2. Methods

The Stanford University Medical Center Institutional Review Board approved this study. The products from our previous study, identified to have the lowest aluminum content, were entered into the calculation engine of our PN software (Infusion Studio; Monterey Medical Solutions Inc., Monterey, CA, USA) for a simulation analysis utilizing our PN patient database. The measured aluminum content of PN solution products can be found in Table 1–Table 3. The products identified as the least contaminated and thus entered into the patient database are are in italics each table. All neonatal patients weighing less than 6 kg who received PN during calendar year 2009 at our institution were included. The total daily aluminum exposure was determined.

**Table 1:** Aluminum content of various products used in parenteral nutrition solutions. Reprinted with the permission from [1].

	Manufacturer (NDC)	Days from Expiry (Range)	Mean Al Content (μg/L)		
			Labeled	Measured (Range)	*p*-Value
Sterile Water	American Regent (0517-3050-25)	1270 (1239-1299)	≤25	<5 (<5)	<0.0001
	American Pharmaceutical Partners (63323-185-50)	511 (356-666)	≤25	5 (5)	<0.0001
	Hospira (0409-7990-09)	584 (507-635)	≤25	6.6 (<5-10)	0.0004
	B. Braun (0264-7850-00)	786 (702-844)	≤25	<5 (<5)	<0.0001
	Baxter (0338-0013-08)	241 (215-270)	25	<5 (<5)	<0.0001
Dextrose 70%	*Hospira* *(0409-7120-07)*	*642 (625-661)*	*25*	*14 (11-16)*	*0.02*
	B. Braun (0264-1290-50)	239 (50-427)	≤25	20 (19-20)	0.001
Amino Acids, TrophAmine^®^ 10%	*Hospira* *(0409-7120-07)*	*642 (625-661)*	*25*	*14 (11-16)*	*0.02*
	B. Braun (0264-1290-50)	239 (50-427)	≤25	20 (19-20)	0.001
Fat Emulsion, Intralipid^®^ 20%	*B. Braun* *(0264-9341-55)*	*579 (539-599)*	*≤25*	*7 (<5-11)*	*0.0008*
	*Fresenius Kabi* *(0338-0519-03)*	*377 (209-507)*	*25*	*11 (<5-17)*	*0.05*
Calcium Gluconate, 100 mg/mL	American Pharmaceutical Partners (63323-311-61)	583 (570-599)	9400	2812 (1969-3495)	0.004
	*American Regent* *(0517-3900-25)*	*416 (415-417)*	*12,500*	*2487 (1928-2887)*	*0.0008*
Magnesium Sulfate 50%	*American Pharmaceutical Partners* *(63323-064-20)*	*234 (81-386)*	*300*	*109 (99-199)*	*0.03*
	Hospira (00409-2168-03)	360 (296-417)	280	122 (103-134)	0.004
	American Regent (0517-2650-25)	552 (537-580)	12,500	165 (113-201)	<0.0001

NDC, National Drug Code; *italics*: least aluminum content and used in patient database.

**Table 2:** Aluminum content of potassium and sodium products used in parenteral nutrition solutions. Reprinted with permission from [1].

Pruducts	Manufacturer (NDC)	Days from Expiry (Range)	Mean Al content (μg/L)		
			Labeled	Measured (Range)	*p*-Value
Potassium Phosphate (3 mmol/mL)	*Hospira* *(0409-4201-01)*	*435 (387-482)*	*51,000*	*4040 (3647-4434)*	*0.005*
	American Regent (0517-2350-25)	290 (203-327)	6,2500	9972 (6512-16,818)	0.004
Potassium Acetate (2 mEq/mL)	*Hospira* *(0409-3294-06)*	*350 (296-417)*	*200*	*22 (11-42)*	*0.003*
	American Regent (00517-2400-25)	495 (368-610)	25,000	744 (521-1120)	<0.0001
Potassium Chloride (2 mEq/mL)	American Pharmaceutical Partners (63323-967-30)	282 (276-387)	100	6.5 (<5-8)	0.01
	*Hospira* *(00409-1513-02)*	*116 (31-174)*	*100*	*5.3 (<5-6)*	*<0.0001*
	American Regent (00517-3450-25)	378 (296-451)	25,000	3242 (3177-3281)	<0.0001
Sodium Phosphate (3 mmol/mL)	*Hospira* *(0409-7391-72)*	*479 (360-568)*	*180*	*29 (17-38)*	*0.001*
	American Regent (00517-3450-25)	378 (296-451)	25,000	3242 (3177-3281)	<0.0001
Sodium Acetate (2 mEq/mL)	*Hospira* *(00409-1513-02)*	*400 (276-478)*	*360*	*73 (54-85)*	*0.0001*
	American Regent (0517-2500-25)	525 (396-610)	25,000	103 (74-138)	<0.0001
Sodium Chloride (2.5 mEq/mL)	Hospira (40mL) (0409-6660-75)	204 (22-386)	100	24 (<5-43)	0.16
	*Hospira (250mL)* *(00409-4219-02)*	*148 (81-215)*	*210*	*8 (<5-11)*	*0.009*
Sodium Chloride (4 mEq/mL)	American Pharmaceutical Partners (63323-088-63)	738 (607-819)	200	52 (50-53)	<0.0001
	*Hospira* *(0409-1130-02)*	*88 (88)* *	*100*	*50* * (50)* *	*NS*

NDC, National Drug Code; *italics*: least aluminum content and used in patient database; * only one lot was sampled for this product.

**Table 3:** Aluminum content of trace elements and multivitamins used in parenteral nutrition solutions. Reprinted with permission from [1].

	Manufacturer (NDC)	Days from Expiry (Range)	Mean Al Content (μg/L)		
			Labeled	Measured (Range)	*p*-Value
Zinc Chloride (1 mg/mL)	*Hospira* *(0409–4090–01)*	*411 (386-451)*	*150*	*11 (5-18)*	*0.0007*
	American Regent (0517-6110-25)	604 (568-635)	2500	249 (54-359)	0.002
Selenium (40 μg/mL)	*American Regent* *(0517–6510–25)*	*481 (386-549)*	*2500*	*285 (106-599)*	*0.005*
Pediatric trace elements	*American Regent Multitrace-4* *(0517–9310–25)*	*518 (518)* *	*2500*	*101 (101)* *	*NS*
	American Regent Pediatric Trace Elements (0517-9203-25)	442 (386-518)	5000	574 (316-739)	0.0009
Pediatric multivitamin	Baxter (54643-5647-0)	261 (239-306)	30	28 (26-29)	0.1
	*Hospira* *(61703–421–53)*	*99 (56-123)*	*42*	*18 (14-25)*	*0.02*

NDC, National Drug Code; *italics*: least aluminum content and used in patient database; * only one lot was sampled for this product.

## 3. Results

The study included 8222 PN patient days in 656 patients. The patients were divided into groups according to their weight in Table 4.

**Table 4:** Patient demographics.

Weight, kg	No. of Patients	No. of Patient Days	Average Parenteral Nutrition Days/Patient	Range of Parenteral Nutrition Days/Patient
0 ≤ weight < 1	69	1112	16.1	1-41
1 ≤ weight < 2	223	2532	11.4	1-70
2 ≤ weight < 3	242	1778	7.3	1-97
3 ≤ weight < 4	194	1784	9.2	1-130
4 ≤ weight < 5	65	666	10.2	1-160
5 ≤ weight < 6	27	350	13	1-173
Total	656	8222	12.5	1-173

We found that 66% of the patient days were among patients weighing less than 3 kg. Premature and small for gestational age infants are the highest risk patient category due to their increased calcium and phosphorus requirements. Aluminum exposure for each weight group is listed in Table 5. The daily Al exposure was highest in neonates weighing less than 1 kg and decreased with the higher weight neonates. On average, the daily Al exposure in our neonatal patients was 10.4 μg/kg/day.

**Table 5:** Measured aluminum exposure.

Patient Weight, kg	Measured Average Aluminum Exposure, μg/kg/day
0 ≤ weight < 1	12.9
1 ≤ weight < 2	11.2
2 ≤ weight < 3	9.2
3 ≤ weight < 4	9.5
4 ≤ weight < 5	9.4
5 ≤ weight < 6	8.8
Total	10.4

Using the least contaminated products, it was still not possible to meet the FDA “safe limit” of less than 4–5 μg/kg/day. Neonates still received 1.8 to 3.2 times the upper limit of allowable aluminum exposure from PN if they received the least contaminated products.

## 4. Discussion

There have been numerous reports of aluminum toxicity from the contamination of PN solutions over the past 3 decades [2,3,4,5,6,7,8,9]. A key study by Bishop et al that contributed to the FDA’s decision to have PN component solutions labeled with their aluminum content compared neurological development in 277 premature infants who received a standard PN formula or an aluminum-depleted PN formula for a period of 5 to 16 days [6]. The median aluminum content in their standard PN, 45 μg/kg/day, was compared with an aluminum-depleted PN solution with an aluminum content of 4 to 5 μg/kg/day. The authors estimated that for infants receiving the standard PN solution, the expected reduction in the Bayley Mental Development Index score would be 1 point per day of parenteral nutrition. A 15 year follow up study of 59 children from these former premature infants examined their bone mineralization [7]. Dual-energy radiograph absorptiometry (DEXA) showed that the now adolescent patients who had received the standard PN solution had lower mineral content and bone area compared to those who had received the aluminum-depleted PN. Their reduced lumbar spine and hip bone mass are potential risk factors for later osteoporosis and hip fractures These findings suggest that total aluminum exposure from prolonged PN is a contributing factor in adverse neurologic and bone development among premature infants. 

Since the FDA modified its regulations in 2000, several studies have demonstrated that manufacturers are not able to meet these stricter regulations [15,16]. This is particularly true in premature infants due to their higher calcium and phosphate requirements compared to adults. A 2006 study calculated the expected daily aluminum exposure from pediatric PN solutions based on the manufacturer-stated aluminum concentration [17]. Even when selecting products reportedly to contain the lowest aluminum concentration, the calculated average aluminum exposure in infants was 59.9 μg/kg/day, exceeding the FDA recommended limit by twelve times the upper limit. The FDA’s recommended limit of 5 μg/kg/day was only feasible in patients weighing over 50 kg. In our 2010 follow-up study, measured aluminum content of compounded PN solutions for neonates was found to be significantly less than the calculated content from the manufacturer’s label [18]. Despite this, aluminum assays of compounded neonatal PN solutions still exceeded the FDA limit of 5 μg/kg/day by 3 to 5 times, (range: 14.9–23.1 μg/kg/day). Our study confirms that by using the least contaminated PN solution products this daily exposure can be reduced to 8.8–12.9 μg/kg/day, which is a 41%–44% reduction from our previous study.

The American Society for Parenteral and Enteral Nutrition Aluminum Task Force published its “Statement on Aluminum in Parenteral Nutrition Solutions” in August 2004 [19]. The Task Force recognized that the FDA regulations only pertain to industry, but acknowledged that safety is the first and foremost consideration. In order to promote safety, the Task Force recommended that all those involved in ordering and preparing PN solutions should be aware of the potential toxicity from aluminum contaminants in the component solutions. The Task Force also recommended that compounding pharmacies may desire to develop a database containing the aluminum content of products used in parenteral nutrition solutions. The Task Force finally recommended that clinicians may want to purchase equivalent products with the lowest aluminum content when possible and should monitor changes in the pharmaceutical market that may affect aluminum concentrations. Our study elucidates another method that can further decrease aluminum exposure in high risk patients. These data can help compounding pharmacies update their practice and insure that the patients are receiving the least aluminum exposure possible with the current products that are available. Health professionals and manufacturers need to continue to develop better methods todecrease the risk of developing aluminum toxicity and eliminating the potential for long-term adverse effects, especially in infants that receive prolonged PN therapy [20,21]. If a purified form of calcium gluconate were available that greatly reduced the amount of Al contamination, it might be possible to meet FDA recommendations. Currently, there are no guidelines for monitoring serum Al levels in patients on long-term PN, but periodic monitoring of Al levels may be indicated in patients with prolonged courses of therapy with high Al exposures [22].

## 5. Conclusion

The actual aluminum exposure in neonates can be significantly decreased when their PN solutions are compounded using the least contaminated solution products. If a concerted effort is made to use the least contaminated products, it may be possible to decrease the potential of developing aluminum toxicity in these vulnerable patients. 
